# Tips for team management

**Published:** 2018-07-31

**Authors:** RD Thulasiraj

**Affiliations:** 1 Executive Director: Aravind Eye Care System, Madurai, India.

**Eye care is effective and efficient when there is a cohesive team effort. This can be achieved by all, by following a few practical steps and having in place simple processes that build an effective team.**

**Figure:**
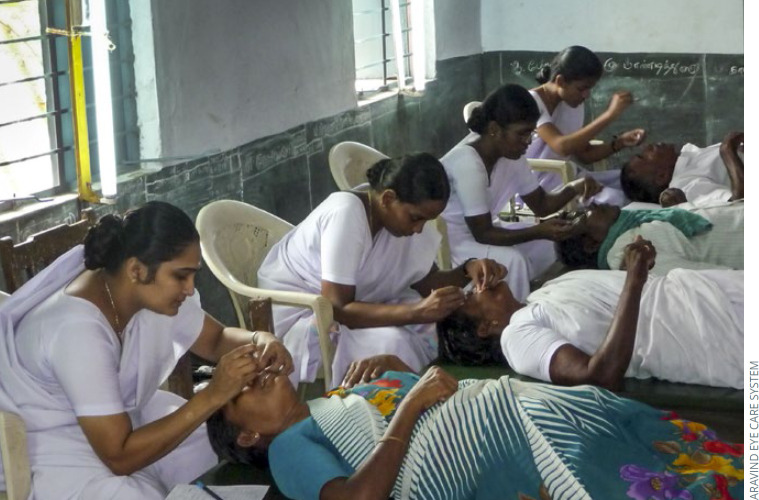
Allied ophthalmic personnel screen for glaucoma. INDIA

Providing eye care is multi-faceted. It starts with working in the community to functions in the hospital such as investigations, diagnosis and treatment. This involves a number of people performing different functions. Thus, by design, eye health requires a team effort. At the same time, specific actions happen at individual levels. The following helps to transform such individual effort into a team endeavour.

**Recruitment and selection:** Every eye hospital has fairly well-defined service area. As much as practical, the recruitment pool should be from this area. In the selection process, while competency is essential, equal importance must be given to the individual's personality. Some of the key elements to be judged are aptitude to work with others, capacity for hard work and being self-driven.

**On boarding:** A new employee becomes productive when he/she integrates with the organisation. This involves developing a deep appreciation of the purpose of institution, knowing the key people and those that he/she will interact with frequently, as well as understanding the system and processes to be followed. It is not a good idea to leave this to chance, or hope that it will happen over time. It is ideal to have this happen within the initial few days. This is possible with a structured orientation programme for all new employees. A good orientation ensures that the employee gets a good understanding of how their work directly relates to the purpose of organisation. This is an important foundation for good teamwork.

**Figure:**
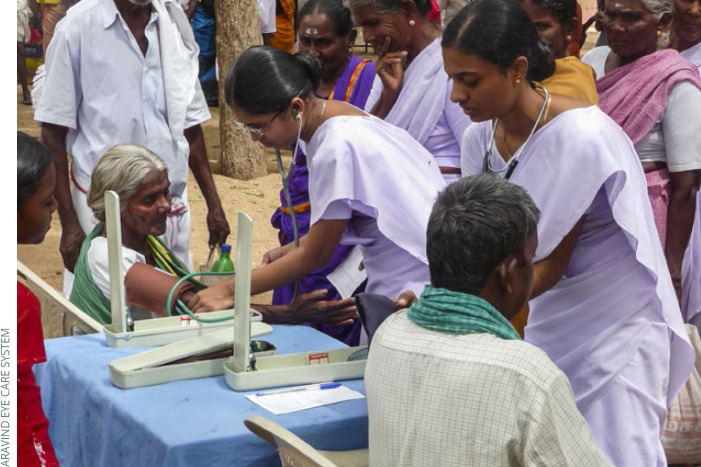
Measuring blood pressure in patients admitted for cataract surgery. INDIA

**Role clarity:** Every employee must have clear understanding of what is expected of them and how their work will be measured or judged. Teams are stronger when staff members have clearly defined roles and know how they contribute to, or impact, the performance of others.

**Enabling work environment:** Staff must have all that is required to perform their role well. This includes basic things like work space, well-functioning instruments or equipment, required supplies and knowing who to approach for issues beyond their current capability. The staffing and other resources provided must fully reflect patient volumes and workload so as to minimise stress, frustration and burn-out.

**Coordination:** While systems and processes can be designed for synergy, in real life things don't happen with clockwork precision. There will be variations in patient volumes, staff availability and challenges with supplies. Periodic coordination meetings and micro-planning for a day or specific tasks are necessary to manage such circumstances.

**Monitoring and continuous learning:** Regular reflection is what leads to continuous improvement through ideas generated by the staff. Often it is this process of seeing one's idea become an activity that builds a sense of belonging and greater motivation to perform well.
